# Mesangial cells from patients with IgA nephropathy have increased susceptibility to galactose-deficient IgA1

**DOI:** 10.1186/s12882-016-0251-5

**Published:** 2016-04-05

**Authors:** Kerstin Ebefors, Peidi Liu, Emelie Lassén, Johannes Elvin, Emma Candemark, Kristina Levan, Börje Haraldsson, Jenny Nyström

**Affiliations:** Department of Physiology, Institute of Neuroscience and Physiology, the Sahlgrenska Academy, University of Gothenburg, Gothenburg, Sweden; Department of Molecular and Clinical Medicine, Institute of Medicine, the Sahlgrenska Academy, University of Gothenburg, Gothenburg, Sweden; Department of Obstetrics and Gynecology, Institute of Clinical Sciences, the Sahlgrenska Academy, University of Gothenburg, Gothenburg, Sweden

**Keywords:** Mesangial cells, IgA nephropathy, PDGF

## Abstract

**Background:**

IgA nephropathy (IgAN) is the most common glomerulonephritis in the world, affecting close to a million people. Circulating galactose-deficient IgA (gd-IgA), present in patients with IgAN, form immune complex deposits in the glomerular mesangium causing local proliferation and matrix expansion. Intriguing though, individuals having gd-IgA deposits in the kidneys do not necessarily have signs of glomerular disease. Recurrence of IgAN only occurs in less than half of transplanted patients with IgAN, indicating that gd-IgA is not the only factor driving the disease. We hypothesize that, in addition to IgA complexes, patients with IgAN possess a subtype of mesangial cells highly susceptible to gd-IgA induced cell proliferation.

**Methods:**

To test the hypothesis, we designed a technique to culture primary mesangial cells from renal biopsies obtained from IgAN patients and controls. The cell response to gd-IgA treatment was then measured both on gene and protein level and the proliferation rate of the cells in response to PDGF was investigated.

**Results:**

When treated with gd-IgA, mesangial cells from patients with IgAN express and release more PDGF compared to controls. In addition, the mesangial cells from patients with IgAN were more responsive to treatment with PDGF resulting in an increased proliferation rate of the cells compared to control. Mesangial cells cultured from patients with IgAN expressed and released more IL-6 than controls and had a higher expression of matrix genes. Both mesangial cells derived from patients with IgAN and controls increased their expressed TGFβ1 and CCL5 when treated with gd-IgA.

**Conclusion:**

We conclude that mesangial cells derived from IgAN patients have a mesangioproliferative phenotype with increased reactivity to IgA and that these cellular intrinsic properties may be important for the development of IgA nephropathy.

## Background

IgA nephropathy (IgAN) is the most common type of glomerular nephritis worldwide. For a long time it has been considered rather benign, but between 30 and 50 % of the patients will eventually develop dialysis-dependent chronic kidney disease [1]. Some patients with morphological signs of IgAN also have IgA vasculitis (IgAV, formerly named Henoch Schönlein purpura) [2, 3]. Patients with IgAN have galactose-deficient IgA (gd-IgA) in their circulation. The gd-IgA easily forms large immune-complexes, which are deposited in the mesangium [4]. These deposits are a key feature of the disease, together with mesangial proliferation and an expanded mesangial matrix, and are used for histopathological diagnosis of IgAN [5]. Several growth factors and cytokines have been suggested to be involved in the proliferation and expansion of the mesangial matrix, most notably PDGF and TGFβ1 [6]. Investigation of PDGFB gene expression in IgAN has shown that there is an increase of PDGFB in patients with IgAN [7], and stimulation of mesangial cells in vitro with PDGF increases their expression of several cytokines and growth factors such as IL-6 and TGFβ1 [8]. IL-6 is a cytokine known to increase in mesangial cells when stimulated with gd-IgA. It has been suggested to be a prognostic marker for IgAN, but its role is still debated [9, 10] Data showing that the complement system (alternative and lectin-pathways) are of importance in IgAN are also accumulating [11]. However, the molecular mechanisms behind the highly variable onset and progression of these glomerular diseases are still elusive. There are familial forms of IgAN [12, 13] where gd-IgA is found in the circulation of asymptomatic relatives. A Finnish investigation of kidneys from suicide or trauma victims revealed that 6.8 % of the kidneys contained IgA depositions although they had no other signs of disease. In a Japanese study of healthy kidney donors, 16.1 % of the investigated kidneys contained IgA deposits [14, 15]. However it is not known if these deposits contain gd-IgA. Indeed, 60 % of transplanted patients diagnosed with IgAN develop deposits in their kidney graft. [16–19] However, the clinical disease recurs in less than half (22–33 %) of the transplanted patients [20–22]. Based on these facts, we wanted to investigate the role of the mesangial cells in development of IgAN. Since there is no specific way to evoke IgAN in cell culture and existing animal models are debated we developed a technique to culture mesangial cells from glomeruli retrieved directly from patient biopsies. Cells were either stimulated with purified IgA1 from IgAN patients (gd-IgA) or with IgA1 from healthy subjects (cIgA). Gene expression levels of cytokines, growth factors and matrix molecules and release of cytokines and growth factors into the cell medium were investigated as well as the proliferative response to PDGF. We were able to demonstrate that mesangial cells from patients with IgAN are significantly more reactive to IgA1 and PDGF stimulation and the cells express and produce higher levels of certain growth factors, cytokines and matrix components.

## Methods

### Patients and controls

This study was conducted in accordance with the declaration of Helsinki and with the approval of the regional ethical review board of Gothenburg. All patients included in the study signed a written informed consent. Six patients with IgA deposits were used as sources of primary mesangial cell cultures (MCs), age 33 ± 15, eGFR 56 ± 27, male to female ratio 5:1, see Table 1. As controls, we used commercially available primary MCs from two healthy donors (Lonza, Basel, Switzerland), age 46 ± 9, male to female 1:1, and cells cultured from a male patient with thin glomerular basement membrane disease (TGBM), age 44 with normal renal function. Three patients with IgAN and three healthy subjects donated 50 ml of blood each used for purification of IgA1.

**Table 1:** Clinical data for patients used for mesangial cell culture

Morphological diagnosis	Sex	Age	Cr-EDTA Clearance	Creat	U-Alb/Creat	Progress (ml/min/year)	BP	BP medication	Immuno-suppression
IgA Nephropathy	M	66	64	98	303	−8.19	138/88	Yes	No
IgA Nephropathy	M	25	21	305	243	−7.54	125/65	No	No
IgA Nephropathy	M	31		214	17	−14.30	115/70	No	No
IgA Nephropathy^a^	M	24	98	80	22	6.84	120/50	Yes	No
IgA Nephropathy^a^	M	24	110	94	11	−0.48	120/70	No	Yes
IgA Nephropathy^a^	F	27		126		−29.37	120/70	No	No

^a^ Clinical diagnosis IgA Vasculitis (Henoch Schönlein Purpura)

### Mesangial cell culture from renal biopsies

**Fig. 1:**
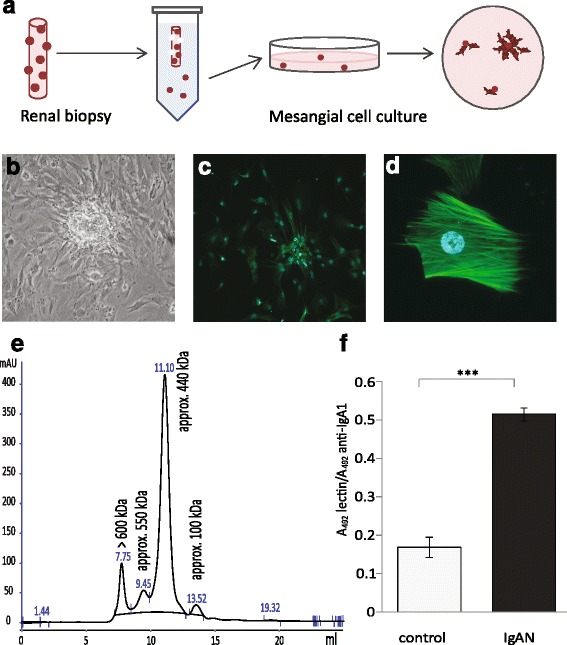
Mesangial cells were cultured from renal biopsies, characterized and treated with IgA. Schematic figure of harvest and culture of mesangial cells (**a**). Mesangial cells growing out from glomeruli (**b**). Immunofluorescence microscopy of smooth muscle actin labeling in mesangial cells. Smooth muscle actin is exclusively localized to the mesangial cells in the glomeruli. Mesangial cells cultured from glomeruli from renal biopsies were stained using an anti-smooth muscle actin antibody (*green*). Nuclei are stained *blue* with DAPI. Magnification 20× (**c**), 40× (**d**). The mesangial cells were negative for endothelial and podocyte markers, see Methods. IgA1 purified from human blood was run on HPLC to detect different sizes of IgA1 (**e**). A representative sandwich ELISA shows that IgA1 purified from patients with IgAN have a higher amount of gd-IgA than IgA1 purified from controls (**f**)

At the time of routine renal biopsy, newly taken biopsies were submerged in ice-cold PBS and loosely attached glomeruli were collected from each biopsy. The glomeruli were transferred by pipetting to cell culture plates coated with attachment factor (Cascade biologics) and cultured in DMEM F12 ham’s medium (Lonza, Basel, Switzerland) supplemented with antibiotics (Lonza) and 20 % human serum. After 10–20 days of incubation, MCs were seen at the edges of the glomeruli that attached to the plate and were subcloned to new cell culture plates without attachment factor. The medium was switched to DMEM F12 supplemented with 20 % FBS (Thermo Scientific, Waltham, MA) and 1 % antibiotics (Lonza). In Fig. 1, the novel technique of harvesting and culturing human mesangial cells from renal biopsies is schematically illustrated (Fig. 1a) and a glomerulus with mesangial cells growing out is shown (Fig. 1b). Cells were identified as MCs by morphology, negative staining for the endothelial marker Ulex Europaeus Agglutinin I (Vector laboratories, Burlingame, Ca) 1:100 and the podocyte marker synaptopodin (Abcam, Cambridge, UK). Positive staining was seen for the mesangial marker smooth muscle actin 1:100 (Abcam), see Fig. 1c and d.

### IgA1 purification

Total IgA1 was isolated from the sera of patients and controls using jacalin conjugated to agarose (Vector) as described previously [23]. The identity of the purified IgA1 was confirmed by HPLC where peaks with monomeric, dimeric and polymeric IgA1 were detected, Fig. 1e. The fraction containing the polymeric IgA1 is often referred to as gd-IgA containing immune-complexes and antiglycan antibodies. To confirm that the ratio of galactose deficient IgA1 was higher in patient samples than control samples a sandwich ELISA using the lectin from *helix aspersa* (Sigma Aldrich, Saint Louis, MO) was used [24]. This confirmed that the IgA1 purified from patients with IgAN had a higher content of galactose deficient IgA1 than the healthy controls, Fig.1f. The concentration of the purified IgA1 was measured using nephelometry.

### Mesangial cell gene expression experiments

Primary MCs obtained from patients with IgAN and controls were cultured in DMEM Ham’s F12 supplemented with 20 % FBS and 1 % antibiotics and used for experiments at passage 5–8. Before treatment all cells were starved in medium containing 0.5 % FBS overnight. Cells were then stimulated with cIgA1 (IgA1 from healthy controls) or gd-IgA1 (IgA1 from patients with IgAN), 100 μg/ml, in starvation medium for 6 h before harvest. Cells were lysed with RLT buffer (Qiagen, Venlo, Netherlands) and RNA was extracted with RNeasy Plus Mini kit using the QIAcube (Qiagen). Concentration and quality of the RNA was determined using the StdSense kit on the Experion (BIO-RAD, Hercules, CA) and/or Nanodrop 2000 (Thermo Scientific). Reverse transcription of the RNA for Q-PCR was performed using High capacity RNA to cDNA kit™ (Applied Biosystems, Foster City, CA). The reaction was carried out using PTC-200 Peltier Thermal cycler (Bio-Rad). The mRNA levels were quantified by real time PCR performed on the ViiA 7 Real-Time PCR system (Applied Biosystems) using custom Taqman® Array 384-well cards (Applied Biosystems) with a gene set of 23 genes in duplicate and GAPDH as housekeeping gene.

### Release of growth factors and cytokines from mesangial cells

Primary IgAN MCs and control MCs were cultured as previously described (passage 5–8) to 80 % confluency and starved overnight and then stimulated with starvation medium containing either cIgA1 or gd-IgA1 (100 μg/ml) for 24 h. Medium was collected and analysed on Bio-Plex 200 system (BIO-RAD) using the 27 human Bio-Plex Pro Assays for cytokines, chemokines and growth factors (BIO-RAD) and run according to the manufacturer's protocol. In addition to IgAN MCs, both mesangial cell cultures from TGBM and primary controls were stimulated for 6 h and the medium analysed with a Bio-Plex for PDGF-BB. Release of TGFβ1 from IgAN MCs after 6 h of stimulation was also analysed using a Bio-Plex for TGFβ1.

### Proliferation assay of mesangial cells

Primary MCs from patients with IgAN or TGBM (control) were seeded in 96 well plates (5000 cells/well, passage 5–8). Cells were starved overnight and then stimulated with 50 ng/ml of either PDGF-BB or PDGF-AB (R&D Systems, Minneapolis, MN) for 20 h before starting the BrdU proliferation assay (Roche) that was run according to the manufacturer's protocol. Incorporation of BrdU was measured using SpectraMax i3 (Molecular Devices, Sunnyvale, CA).

### Statistics

For gene expression studies and release of cytokines one way Anova with multiple comparisons with Bonferroni correction was used. For statistics of proliferation rate and ratio of gd-IgA student’s *t*-test was used. Data is presented as mean ± SEM except for results from proliferation experiments that are presented as min and max. P < 0.05 was considered statistically significant.

## Results

### IgAN mesangial cells exhibit gd-IgA induced PDGF expression and increased sensitivity to PDGF

**Fig. 2:**
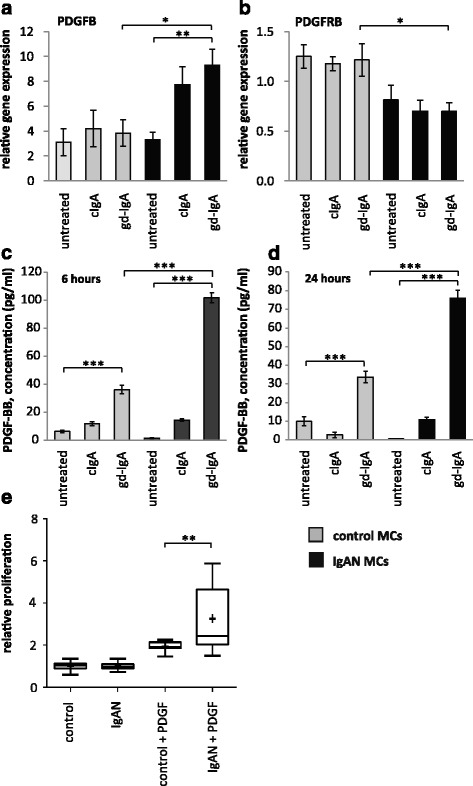
PDGF is the major growth factor for mesangial cells we therefore wanted to investigate the expression of PDGF in mesangial cells from controls or patients with IgA deposits as well as their response to stimulation with PDGF. Mesangial cells (MCs) from patients with IgA nephropathy (IgAN) and controls were stimulated with IgA1 purified from blood from healthy controls (cIgA) or patients with IgAN (gd-IgA) or medium only for 6 h and the gene expression was investigated. The gene expression of PDGFB was not affected for the control MCs when stimulated with either cIgA or gd-IgA. The gene expression of PDGFB was increased for the IgAN MCs when stimulated with either cIgA or gd-IgA and stimulation of gd-IgA on IgAN MCs had a significantly higher expression than in control MCs stimulated with gd-IgA (**a**). In contrast the gene expression of PDGFRB (gene coding for platelet-derived growth factor receptor beta) was lower in all samples from IgAN MCs compared to the control MCs, but the gene was not further affected by stimulation with either cIgA or gd-IgA (**b**). Medium was collected from IgAN MCs and control MCs after 6 and 24 h of stimulation with cIgA and gd-IgA and the release of PDGF-BB into the medium was measured. Both the IgAN MCs and control MCs released the highest amounts of PDGF-BB when stimulated with gd-IgA, but the levels was significantly higher for the IgAN MCs than for the control-MCs after 6 h (**c**) and 24 h (**d**). Mesangial cells from patients with IgAN and control were treated with 50 ng of either PDGF-AB or -BB or medium only and the relative proliferation of the cells was measured. Stimulation of the IgAN MCs with PDGF gave a significant increase of the relative proliferation compared to control MCs (**e**). *Grey bars* represent control MCs, *black bars* represent IgAN MCs *P < 0.05, **P < 0.01, **P < 0.001. Error bars in (**a**), (**b**), (**c**) and (**d**) represent SEM and in D min to max, + represents mean

IgAN patients have shown increased PDGFB expression [7] and we thereby investigated if PDGFB expression was altered in IgAN MCs following stimulation with gd-IgA. We found that a 6 h stimulation with gd-IgA significantly increased the gene expression of PDGFB (gene coding for platelet derived growth factor subunit B) compared to untreated IgAN MCs, see Fig. 2a. The control cells did not alter their gene expression of PDGFB in response to either treatment. The gene expression of the receptor PDGFRB (gene coding for platelet-derived growth factor receptor beta) was not affected when stimulated with IgA in any of the cells, and the expression of the receptor was significantly lower in the IgAN MCs than in the control MCs independent of treatment, see Fig. 2b. To confirm the altered expression on protein level, PDGF levels was investigated by measuring the release of PDGF-BB into the cell culture medium after 6 and 24 h of stimulation with cIgA or with gd-IgA, Fig. 2c and d. Both the IgAN MCs and control MCs responded with significant increases in the release of PDGF-BB when stimulated with gd-IgA for 6 or 24 h but the amount released by the IgAN MCs after gd-IgA stimulation was significantly higher than the amount released by the control MCs. In addition, similar results as in Fig. 2c and d were obtained using the control mesangial cells from a patient diagnosed with TGBM. Significant differences were observed between untreated and gd-IgA for both groups of cells (control; untreated 10.5 ± 0.1, gd-IgA 29.9 ± 4.5, *P* < 0.001, IgAN; untreated 0.72 ± 1.8, gd-IgA 58.0 ± 3.8, *P* < 0.001). There was also significantly higher release of PDGF-BB for IgAN MCs treated with gd-IgA compared to TGBM MCs (*P* < 0.001).

To investigate how the mesangial cells from IgAN patients responded to the increase in PDGF synthesis and release, cells were stimulated with PDGF-AB and BB and a proliferation assay was performed using BrdU (Fig. 2e). Interestingly, the IgAN MCs were more reactive in their proliferative response to PDGF than the control cells (TGBM). These results show that mesangial cells derived from patients with pathogenic IgA deposits both produce more PDGFB and are more sensitive to PDGFB than control MCs.

### IgA increases IL-6 expression in mesangial cells

**Fig. 3:**
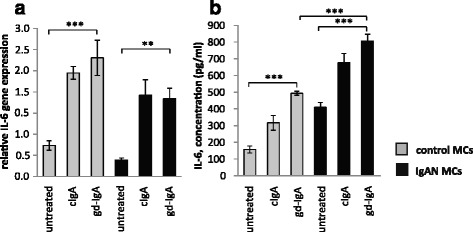
Gene expression and release of IL-6. IL-6 expression is known to increase in mesangial cells when stimulated with gd-IgA and has been suggested as a prognostic marker for IgAN but its role is still debated. Mesangial cells (MCs) from patients with IgA nephropathy (IgAN) and controls were stimulated with IgA1 purified from blood from healthy controls (cIgA) or patients with IgAN (gd-IgA) or medium only for 6 h and the gene expression was investigated. Stimulation with IgA on both controls MCs and IgAN MCs increased the gene expression of IL-6 in a similar pattern (**a**). The release of IL-6 into the medium after 24 h of stimulation was most pronounced for the gd-IgA treatment for both groups of cells, but the IgAN MCs released significantly more IL-6 than the control MCs stimulated with gd-IgA (**b**). *Grey bars* represent control MCs, *black bars* represent IgAN MCs * *P* < 0.05, ** *P* < 0.01, ***P < 0.001, error bars represent SEM

Since gd-IgA increases PDGF expression in IgAN MCs, which from previous studies is known to increase IL-6 in mesangial cells [8] we tested if gd-IgA could increase IL-6 in IgAN MCs. IgAN MCs and control MCs were stimulated with gd-IgA or cIgA. All of the IgAs tested resulted in a significant increase of IL-6 gene expression in IgAN MCs and controls, Fig. 3a. In addition, to test if the IL-6 secretion from IgAN MCs cells was altered when compared to control MCs, release of IL-6 into the medium was analysed and both IgAN MCs and control MCs were shown to release significantly more IL-6 when stimulated with gd-IgA. Interestingly, the release of IL-6 was significantly higher in the IgAN MCs in response to gd-IgA than in the control MCs, see Fig. 3b.

### The gene expression of TGFB1 is increased in mesangial cells when exposed to gd-IgA

**Fig. 4:**
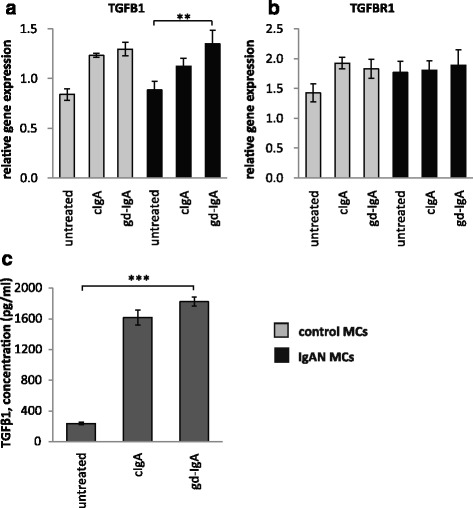
Gene expression of TGFB1 and TGFBR1 and release of TGFβ1. Mesangial cells (MCs) from patients with IgA nephropathy (IgAN) and controls were stimulated with IgA1 purified from blood from healthy controls (cIgA) or patients with IgAN (gd-IgA) or medium only for 6 h and the gene expression was investigated. Stimulation of control MCs and IgAN MCs with gd-IgA gave a significant increase in the gene expression of TGFB1 (gene coding for TGFβ1) (**a**). The gene expression for TGFBR1 (gene coding for TGF-beta receptor type-1) was not significantly affected by any of the treatments (**b**). IgAN MCs were stimulated with either cIgA or gd-IgA for 6 h and the release of TGFβ1into the cell culture medium was investigated. Both treatment with cIgA and gd-IgA resulted in an increased release of TGFβ1 compared to untreated cells (**c**). *Grey bars* represent control MCs, *black bars* represent IgAN MCs * *P* < 0.05, ** *P* < 0.01, ***P < 0.001, error bars represent SEM

PDGF has previously been shown to increase the expression of the growth factor TGFβ1 in mesangial cells, and by doing so induce an expansion of the mesangial matrix [6]. We therefore investigated if MCs exposed to gd-IgA increased the gene expression of TGFB1 (gene coding for TGFβ1) both in IgAN MCs and control MCs and compared to their respective untreated control, Fig. 4a. We found that the gene expression of TGFB1 was significantly increased in IgAN MCs after treatment with gd-IgA, and the same trend was found in the controls but not to significant levels. The expression of the receptor TGFBR1 was not significantly affected by stimulation with any IgA1 in either group of cells, see Fig. 4b. We also measured the release of TGFβ1from the IgAN MCs and found that treatment with either cIgA or gd-IgA increased the release of TGFβ1 into the cell culture medium, see Fig. 4c.

### gd-IgA increased the release of CCL5 (RANTES) by mesangial cells

**Fig. 5:**
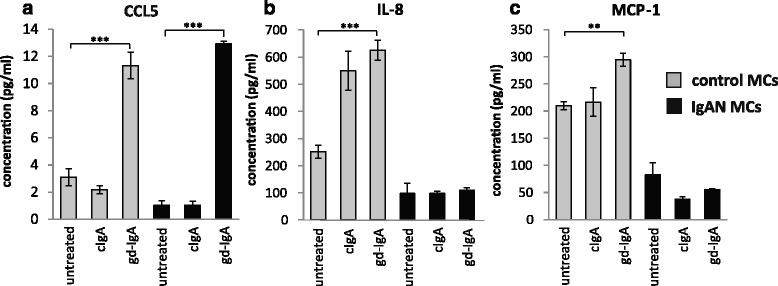
Release of CCL5, IL-8 and MCP-1. Mesangial cells (MCs) from patients with IgA nephropathy (IgAN) and controls were stimulated with IgA1 purified from blood from healthy controls (cIgA) or patients with IgAN (gd-IgA) or medium only for 24 h and the release of cytokines into the medium was investigated. Stimulation of control MCs and IgAN MCs with gd-IgA resulted in an increased release of CCL5 in both groups of cells, in contrast stimulation with cIgA did not affect either group of cells at all (**a**). Only the control MCs responded to treatment with gd-IgA with an increased release of IL-8 (**b**) and MCP-1 (**c**). *Grey bars* represent control MCs, *black bars* represent IgAN MCs * *P* < 0.05, ** *P* < 0.01, ***P < 0.001, error bars represent SEM

It has been reported that the level of proteinuria in patients with IgAN correlates to intrarenal levels of CCL5 (also known as RANTES) [25]. To investigate if CCL5 levels are increased in IgAN MCs the release of CCL5 was studied in the presence of gd-IgA and cIgA. A pronounced increase of CCL5 was found in both IgAN MCs and control MCs following gd-IgA exposure and conversely cIgA did not trigger a CCL5 response, Fig. 5a. In contrast, other chemokines such as IL-8 and MCP-1 was shown to be elevated solely in control MCs already at baseline with increased levels following gd-IgA incubation, see Fig. 5b and c. Intriguingly, the IgAN MCs did not change their release of these cytokines either in response to cIgA or gd-IgA. The other cytokines, chemokines and growth factors included in the assay (see manufactures information about Bio-Plex Pro Human 27-plex, BIO-RAD) were either expressed at very low levels or/and did not respond to stimulation with cIgA nor gd-IgA for any of the mesangial cells.

### IgAN MCs have an increased expression of matrix-associated genes compared to controls

**Fig. 6:**
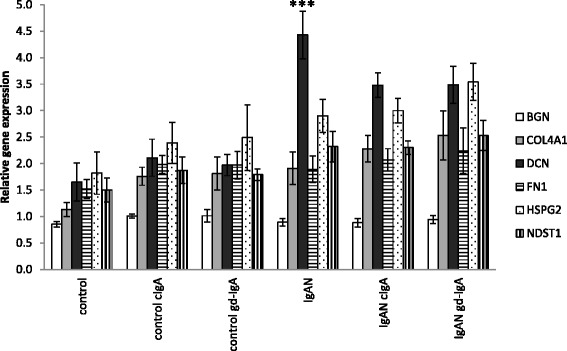
Gene expression of matrix associated genes. One of the main findings in IgAN is expanded mesangial matrix and mesangial proliferation. In order to investigate if cells from patients with IgA-depositions in the kidney express more matrix associated genes we investigated the gene expression of selected matrix genes, namely; BGN (biglycan), COL4A1 (collagen alpha-1(IV) chain), DCN (decorin), FN1 (fibronectin), HSPG2 (perlecan) and NDST1 (heparan sulfate N-deacetylase/N-sulfotransferase 1). The expression of DCN (a small proteoglycan) was significantly higher in untreated IgAN MCs than controls. Overall we could see a trend that IgAN MCs had a higher expression of matrix genes, in the unstimulated groups, and that stimulation with cIgA and gd-IgA increased the expression for all groups. * *P* < 0.05, ** *P* < 0.01, ***P < 0.001, error bars represent SEM

To further study alteration in matrix components in IgAN MCs, the expression of the matrix genes: BGN (biglycan), COL4A1 (collagen alpha-1(IV) chain), DCN (decorin), FN1 (fibronectin), HSPG2 (perlecan) and NDST1 (heparan sulfate N-deacetylase/N-sulfotransferase 1) was analysed and found to be elevated in IgAN MCs when compared to control MCs. The elevation of matrix genes was still found to be present in IgAN MCs when cIgA and gd-IgA was added to the cultures, see Fig. 6.

## Discussion

In this study, we have shown that it is possible to harvest and to culture primary mesangial cells from renal biopsies patients with for example IgAN and to study those cells in vitro. Our hypothesis was that the mesangial cell (MC) from patients with IgAN are hyperresponsive to gd-IgA and more prone to proliferation than mesangial cells from individuals without kidney disease. The results obtained support our hypothesis and may at least partly explain why renal IgA deposits only develop into nephropathy in a subset of the human population. It is noteworthy, that the phenotype of the IgAN MC display only subtle differences compared to the MCs from healthy controls, which is fitting for a disease that progresses slowly over years or even decades.

As outlined in the Introduction, IgAN recurs histologically in at least 60 % of transplanted patients [16] and it develops into clinically significant nephropathy in 20–35 % of all transplanted IgAN patients [20–22]. In a study of 38 kidney transplant patients with recurrence of IgAN, 22 without and 17 healthy controls, the risk of recurrence correlated with the levels of the following three biomarkers: gd-IgA, IgG autoantibodies against gd-IgA and soluble CD89 [26]. However, it is still unknown how these biomarkers may be involved in the onset and progression of IgAN.

In order to further understand the mechanisms behind onset of IgAN we developed a technique to culture primary mesangial cells from human renal biopsies since there is a lack of good in vitro and in vivo models for the disease. Culturing mesangial cells from biopsies is not trivial since the renal tissue is sparse and the glomeruli are badly needed for the morphological diagnosis. It is known however, that with each needle biopsy, there are 3–6 glomeruli that are cut loose. By dipping the biopsy needle in cold saline before processing the biopsy tissue core, these glomeruli (that otherwise would be wasted) can be harvested and placed (cut or uncut) on Petri dishes. Using proper culturing media, mesangial cells can be selected isolated and cultured. Since these cells are primary cultures in a low passage, they are likely to maintain their distinct phenotype. The drawback is that primary clones will stop growing and/or change their phenotype after a finite number of passages compared to for instance immortalized cells. Another limitation is that it is not possible to culture mesangial cells from all patients, which may introduce a selection bias. A third limitation is that active inflammation may alter the glomerular mesangial cell phenotype per se. It is however likely that the latter effect is “washed out” during the inflammatory-free culturing process hence restoring the mesangial cell to its “control state”.

We investigated gene and protein expression of several known inducers of proliferation and matrix expansion in mesangial cells from IgAN patients and from controls. We also conducted functional assays to assess proliferation of these cells with and without stimulation with IgA purified from patients diagnosed with IgAN (gd-IgA) or from healthy controls (c-IgA). Stimulation of mesangial cells from patients with IgAN with gd-IgA significantly increased the gene expression of PDGFB, while the control MCs did not. Moreover, IgAN MCs released significantly more PDGF-BB than control MCs when stimulated with gd-IgA confirming the gene expression data. PDGF-BB has a proliferative and matrix expanding effect on mesangial cells both in vitro and in vivo [27, 28], and such an autocrine effect has previously been established for MCs. However, not only did the IgAN MCs produce more PDGF, the cells responded to PDGF stimulation with a higher proliferation rate than control MCs. Thus, IgAN MCs are more sensitive to gd-IgA stimulation, produce more PDGF and are more sensitive to PDGFB. These intrinsic properties of the IgAN MCs contribute to the increased proliferation and matrix expansion that is a hallmark of IgAN.

TGFβ1 is another growth factor known to be involved in IgAN and shown to be up-regulated in IgA treated mesangial cells [29]. We could confirm the finding of Lai et al. in IgAN MCs as well as in control MCs. IL-6 has also been suggested to be a part of the pathogenesis of IgAN and gd-IgA has been shown to increase the gene expression of IL-6 [23], but the effect of cIgA on mesangial cells IL-6 expression was not investigated in that study. Mesangial cells also produce CCL5 [30], and the levels of intrarenal gene expression of CCL5 seem to correlate to the level of proteinuria in patients with IgAN [25]. In our hands, all mesangial cells (IgAN and control) increase their expression either on the gene or protein level of TGFβ1, IL-6 and CCL5 in response to cIgA and gd-IgA. In contrast MCP-1 and IL-8 were only upregulated in the control MCs. We hypothesise that TGFβ1, IL-6 and CCL5, are not part of the unique MC phenotype of IgAN patients, but rather constitute a general MC response to IgA.

Among the investigated matrix genes we only found a significant upregulation of the gene expression for decorin but could see a clear trend that IgAN derived MCs have an increased level of expression of matrix genes. In an animal model of glomerulonephritis that is induced by injection of anti-thymocyte serum in rats Okuda et al. showed that increased TGFβ1 expression was coupled to increased production of proteoglycans and glomerular matrix accumulation [31] and involvement in IgAN has been suggested by us and others [32, 33]. Decorin is a small proteoglycan known to be a natural inhibitor for TGFβ1 and in previous studies we have shown that both TGFβ1 and decorin have increased gene expression in glomeruli from patients with IgAN [33]. In that study, we showed that the proteoglycan perlecan (gene HSPG2) is up-regulated in glomeruli from patients with IgAN, both on the mRNA and the protein level. Heparan sulfate proteoglycans can bind several growth factors, including PDGF-BB for which the interaction governs tissue distribution and possibly also the bioavailability of the growth factor [34, 35]. In this paper, however, the increase in perlecan gene expression did not reach statistical significance.

### Mechanisms behind IgA nephropathy – the triple hit hypothesis

The results of the present study give us confidence to propose a revision of the current theory of how IgAN develops and progresses. The first requirement is the presence of gd-IgA and IgG autoantibodies against IgA forming IgA-complexes that forms deposits in the mesangium of the kidneys. Second, the individual must have mesangial cells with a IgAN-prone phenotype, i.e. increased production of PDGF and increased sensitivity to PDGF and to gd-IgA. Thirdly, mucosal infections (respiratory or intestinal) promote production of IgA and trigger reactions including complement alternative and/or lectin pathway alterations [11]. The latter can explain both the chronic low-grade mesangial inflammation and the acute exacerbations with (macroscopic) hematuria, proteinuria and temporarily elevated serum creatinine concentrations.

## Conclusion

In conclusion, the mesangial cells of patients with IgA nephropathy have a specialized phenotype and are more reactive to PDGF and gd-IgA stimulation. The cells also produce more PDGF. We believe one can say that at least three factors are needed to develop IgAN; circulating IgA-complexes, activation of the complement system [11], and a mesangial cell phenotype prone to respond to IgA stimulation with proliferation and matrix production.

### Ethics

This study was conducted in accordance with the declaration of Helsinki and with the approval of the regional ethical review board of Gothenburg, Sweden.

### Consent

All patients included in the study signed a written informed consent before participating.

### Availability of supporting data

No data has been submitted to any open access databases. All data supporting the study is presented in the manuscript or available upon request.

## Abbreviations

gd-IgA: galactose deficient Immunoglobulin A
IgA1: Immunoglobulin A subclass 1
IgAN: IgA nephropathy
IgAV: IgA vasculitis
MCs: mesangial cells
TGBM: thin glomerular basement membrane disease
